# Study of the Influence of Chitosan-Wrapped Carbon Nanotubes on Biopolymer Film Properties

**DOI:** 10.3390/polym17070889

**Published:** 2025-03-26

**Authors:** Aurora G. Magallanes-Vallejo, Ana B. López-Oyama, Eugenio Rodríguez González, Deyanira Del Angel-López, Eder U. Pulido-Barragán, Crescencio García-Guendulain, Tomás J. Madera-Santana, César Rodríguez-Beas, Rogelio Gámez-Corrales

**Affiliations:** 1 Instituto Politécnico Nacional, Centro de Investigación en Ciencia Aplicada y Tecnología Avanzada-Unidad Altamira, Km 14.5 Carr, Tampico-Puerto Industrial, Altamira 89600, Tamaulipas, Mexico; aumagallanes@gmail.com (A.G.M.-V.); ddelangel@ipn.mx (D.D.A.-L.); eder.pulido.bar@gmail.com (E.U.P.-B.); 2 Departamento de Investigación en Física (DIFUS), Universidad de Sonora, Blvd. Transversal S/N, Hermosillo 83000, Sonora, Mexico; 3 Secihti-DIFUS, Universidad de Sonora, Blvd. Transversal S/N, Hermosillo 83000, Sonora, Mexico; 4 School of Engineering and Science, Tecnologico de Monterrey, Mexico City 14380, Mexico; crescencio.garcia@tec.mx; 5 Centro de Investigación en Alimentación y Desarrollo, A.C., Carr. Gustavo E. Astiazarán Rosas No. 46, Col. La Victoria, Hermosillo 83304, Sonora, Mexico; madera@ciad.mx; 6 Departamento de Física, Universidad de Sonora, Blvd. Transversal S/N, Hermosillo 83000, Sonora, Mexico; cesar.rodriguez@unison.mx (C.R.-B.);

**Keywords:** chitosan-wrapped, carbon nanotubes, film-forming properties, bioengineering

## Abstract

Due to their biocompatibility and non-toxicity, biopolymer-based films hold significant importance in bioengineering. It is imperative to comprehend the influence of chitosan molecular weight and filler materials nature on the crystalline structure and their subsequent effect on film properties. The aim of this research was to determine how carbon nanotubes embedded within chitosan can significantly improve the performance of biopolymer-based films produced by the solvent-casting technique. Four probe measurements demonstrated that films of medium-molecular-weight chitosan/carbon nanotubes displayed an electrical conductivity value of 0.0132 S cm^−1^, resulting in films with a low sheet resistance value of 0.0156 mΩ/Υ. Based on XRD findings, it has been demonstrated that films containing carbon nanotubes have shifted the (002) plane of chitosan towards higher angles, favoring chitosan crystal form II, which could be responsible for the enhanced mechanical performance. Structural characteristics, such as lattice strain (e), grain size (D), and dislocation density, have been calculated using the Williamson–Hall method, in which the medium-molecular-weight chitosan/CNTs film samples displayed the best crystalline quality. SEM images revealed nanotube diameters ranging in size from 140 to 300 nm, suggesting that the chitosan was effectively wrapped along carbon nanotubes. Our results indicate that developing chitosan-wrapped carbon nanotube films introduces them as potential materials for bioengineering and biomedical research.

## 1. Introduction

The emergence of advanced materials allows for the combining of properties of their individual constituents at a molecular level. Carbon nanotubes (CNTs) have demonstrated novel prospects for developing electrically conductive materials with adequate optical, electrical, mechanical, and antimicrobial properties. These materials exhibit remarkable compatibility with biopolymers, such as chitosan (Cs), which is readily accessible, affordable, and environmentally friendly. Therefore, they can be employed to produce Cs-based films, which have potential applications in the field of bioengineering. In the pursuit of producing films containing chitosan–carbon nanotubes with high precision and selectivity, it is imperative to preserve individual characteristics, such as biocompatibility and high electrical conductivity, along with adequate mechanical and optical performance.

Cs is a polysaccharide biopolymer derived from the deacetylation of chitin and is composed of deacetylated units or copolymers of D-glucosamine and N-acetyl-D-glucosamine [1]. The degree of deacetylation (DDA) is determined by the molar ratio of deacetylated units along the polymer stiff backbone. Cs is a highly attractive film-forming material due to its biodegradability, non-toxicity, biocompatibility, and ability to form flexible, durable, and functional films. Contrary to many synthetic polymers, it is derived from a renewable, natural resource, and its antimicrobial properties and ease of modification make it ideal for a wide range of applications, from food packaging to biomedical devices and pharmaceuticals over other synthetic alternatives [2]. Cs has many interesting properties, such as its ability to form gels and transparent solutions that solidify into clear films upon drying. To improve it, it has been reinforced with nanocrystals [3], quantum dots [4], nanoparticles [5], and reduced graphene oxide [6,7,8,9]. The amphiphilic nature of Cs induces it to coil along the carbon nanotube surface, exposing its functional groups. This resulted in a better distribution and performance of CNTs within the polymer matrix [10]. The behavior is attributed to the extensive availability of amino and hydroxyl functional groups in Cs, which possess the ability to promote an attractive interaction with defect sites or functional groups onto CNTs, resulting in wrapped chitosan along their surface. Rungrotmongkol et al. employed an MD simulation approach to study the improvement of dispersion and solubility of single-walled carbon nanotubes (SWCNTs) into Cs with a 60% DDA. The authors reported theoretical calculations about the aggregation behavior attributed to hydrophobic and van der Waals interactions between the aromatic rings of CNTs, as well as the charge-charge repulsive force between the ammonium and acetyl groups of Cs-wrapped on carbon nanotubes. This allows for a facile association as well as increases the dispersion without modifying CNTs properties [11,12,13]. Cs-based films offer interesting challenges and opportunities due to their inherent properties, such as the DDA and molecular weight, which influence the film properties. Despite decades of research on Cs, there has been scarce investigation into the role of the Cs molecular weight in the wrapping of carbon nanotubes. A thorough evaluation of the wrapping of three-different-molecular-weight chitosan around CNTs may provide confirmation of the effectiveness of the polymer coating.

Cs-wrapped carbon nanotubes have garnered significant attention in biomedical applications due to attractive characteristics, including biocompatibility and non-toxicity, as well as their potential for integration with biological systems [14]. Further, the Cs wrapped along CNTs lead to an improvement in the flexibility and strength of the films by maintaining structural integrity. However, future research will focus on improving the synthesis and functionalization of carbon nanotube composites to further increase their biocompatibility and performance by exploiting the advantages of Cs-based films. For biomedical applications, Cs-based films must possess adequate mechanical strength and flexibility because a low tensile strength value can lead to premature failure under physiological conditions. Therefore, the incorporation of filler materials or structural modifications are required to improve these properties. The research of diverse strategies, such as the production of composite materials for enhancing the mechanical performance of Cs-based films, and the reduction in water sensibility for improving cell interaction, are challenges to overcome. These ongoing initiatives aim to enhance the efficacy of chitosan-based films for biomedical purposes, thereby addressing their inherent limitations. The incorporation of carbon-based materials, nanoparticles, and other materials had a significant impact on chitosan crystallinity, resulting in favorable biopolymer film performance [15]. However, research into the influence of carbon nanotubes on the long-term effectiveness of biopolymer films is of great importance.

The experimental methods for producing biopolymer composite films of controlled size and shape include several techniques designed to adjust their properties for specific applications. These methods include electrospinning, extrusion, sol–gel processes, freeze-drying, in situ polymerization, and solvent casting. Electrospinning produces nanofiber systems with high surface area and tunable diameters. This makes them ideal for applications requiring a high surface-to-volume ratio. Extrusion, on the other hand, is commonly used to produce biopolymer composites into shapes such as sheets or films but often lacks the precise control of film morphology and thickness required for certain applications. The sol–gel process allows fine control over the microstructure and porosity of thin films but is generally more complex and is mainly applied to the production of transparent films rather than biopolymer composites. Lyophilization or freeze-drying is effective in maintaining the structural integrity of composites with controlled porosity. However, it does not provide the same precision in controlling film thickness as other methods. Of these techniques, solvent casting is the most effective. It provides precise control of film thickness, morphology, and uniformity [16]. This makes it particularly advantageous to produce biopolymer composite films with unique properties.

The addition of CNTs further enhances chitosan-based film properties, reinforcing the structure and improving electrical conductivity and mechanical performance. Hydrated and anhydrous crystal forms of chitosan have demonstrated improved properties when combined with CNTs, with hydrated chitosan generally offering superior film formation, mechanical performance, and low resistivity values. These enhanced properties make chitosan, in both hydrated and anhydrous forms, suitable for applications in bioengineering. The wrapping behavior of chitosan around CNTs is crucial to these outcomes, as it directly influences the mechanical strength, stability, and electrical performance of the composite material. This article aims to investigate the influence of the molecular weight of chitosan on the effective wrapping of carbon nanotubes in biopolymer films obtained by the solvent evaporation technique. Studying these properties can contribute to the production of biopolymer films, where present and future challenges can be considered to optimize the development of films that significantly broaden their scope of applications in fields such as bioengineering.

## 2. Materials and Methods

### 2.1. Materials and Film Samples Preparation

Powdered chitosan with a DDA of 75% and three distinct molecular weights for low-, medium-, and high-molecular-weight chitosan, namely LMW-Cs (50 kDa), MMW-Cs (190 kDa), and HMW-Cs (310 kDa), respectively, as well as multiwalled carbon nanotubes, HCl, NaCl, and acetic acid, were purchased from SIGMA-Aldrich, St. Louis, MO, USA. The reagents were analytically graded and used as received without further purification.

The preparation of the film samples was performed in two steps. The initial step involved the preparation of pure chitosan liquid solutions by dissolving each chitosan sample in aqueous acetic acid (2.0%) to obtain LMW, MMW, and HMW chitosan liquid solutions. For further details, refer to the Supplementary Information (Figure S1a,b). The second step was conducted to prepare the Cs/CNTs solution by dissolving MWCNTs powder into the acid solution prepared in step one in a 5:1 Cs/CNTs ratio (*v*/*w*) to produce chitosan/CNTs liquid solutions consisting of LMW-Cs/CNTs, MMW-Cs/CNTs, and HMW-Cs/CNTs solutions, respectively. Each final liquid solution for both pure chitosan and chitosan/carbon nanotubes was processed using an Ultra-Turrax homogenization unit for 10 min at 8000 rpm and then allowed to equilibrate for 24 h. The liquid solutions were used to carry out the optical and microbiological analyses described in the Supplementary Information file. The second step followed the procedure reported by González-Martínez et al. [6]: an appropriate amount of Cs and Cs/CNTs solution was placed into Petri dishes and allowed to evaporate the solvent for 72 h at 30 °C inside an oven. All films were produced and characterized at room conditions and relative humidity of ≈21%, typical of Hermosillo, Sonora, México city [29°05′56″ N 110°57′15″W].

### 2.2. Film Samples Characterization

#### 2.2.1. Fourier Transform Infrared (FTIR) Spectroscopy

FTIR spectra were obtained by employing a Thermo Scientific FTIR instrument, the Nicolet 5s model (Madison, WI, USA), equipped with an ATR iD3 accessory. A total of 64 scans with a resolution of 4 cm^−1^ were acquired within the range of 600 to 4000 cm^−1^.

#### 2.2.2. Thickness

Pure Cs and Cs/CNTs film thickness were measured using a micrometer (Mitutoyo, Kawasaki, Japan) with 0.01% precision. The average value was determined by taking measurements in five different zones.

#### 2.2.3. Ionic Exchange Capacity (IEC)

Each film sample was subjected to an IEC analysis in triplicate following the titration method. The initial activation of each film sample consisted of the alternating conversion of the pure Cs and Cs/CNT films to their H^+^ and OH^−^ forms. For 48 h, the film samples were exposed to a solution of 0.1 M HCl and 0.1 M NaCl.

#### 2.2.4. Swelling and Solubility

The degree of swelling (DS) and solubility in water (SW) of each sample film was calculated following the methodology reported by Rodriguez et al. [17]. To achieve equilibrium, the film samples were immersed for one hour in distilled water at room temperature. The percentage of the degree of swelling (%DS) was calculated with the next equation:

(1) $$\%DS=\frac{w_{e}-w_{0}}{w_{0}}\times \;100$$

The initial dry weight of a film is denoted as *w*_0_, while *w*_e_ indicate the weight of the films after the swelling process has taken place. After 24 h, the swelled Cs and Cs/CNTs film samples were then dried at 60 °C. The percentage of solubility in water was calculated using this equation:

(2) $$\%SW=\frac{w_{0}-w_{d}}{w_{0}}\times 100$$

where *w*_0_ is the dry initial weight of the films, and *w*_d_ is the film dry weight after the drying process. DS and SW measurements were performed in triplicate.

#### 2.2.5. XRD Analysis

A Bruker D8 Advance X-ray diffractometer with CuKa radiation was employed to analyze the X-ray diffraction patterns of the film samples. The wavelength of the X-ray beam was 0.15406 nm. The data were collected at a scanning rate of 2° per minute over a range of scattering angles (2θ) from 10 to 60°.

#### 2.2.6. X-Ray Photoelectron Spectroscopy (XPS)

The technique was suitable to analyze the surface of selected film samples of MMW-Cs and MMW-Cs/CNTs using a PHI 5000 Versaprobe instrument, Chanhassen, MN, USA. The high-resolution spectra of the elements detected were collected by using monochromatic MgKa radiation as the X-ray source.

#### 2.2.7. Mechanical Characterization

An evaluation of the tensile strength (TS), elongation-at-break (EB), modulus of toughness, and Young’s modulus of Cs/CNTs specimens was conducted using a texturometer (Texture Technologies Corp., New York, NY, USA) based on the ASTM D882 standard method [18]. The testing was performed at room temperature and ambient conditions of 25 °C and 21% relative humidity. Each strip from film samples was cut into 10 mm wide, 40 mm long, and 23 mm longitudinal slits with a razor blade. For experimental purposes, a crosshead speed of 10 mm/min and a gauge length of 30 mm were used. Five replicates of the mechanical test were performed for each film.

#### 2.2.8. SEM Analysis

A JEOL JSM-7800F (Tokyo, Japan) was employed to obtain high-resolution SEM images of the MMW-Cs/CNTs film at 5.0 eV.

#### 2.2.9. 4-Probe Measurements

The film samples were tested for resistivity with a source measurement unit with a 0.40-inch equidistant tip configuration (Keithley Model 4200-SCS system, Solon, OH, USA). A gate voltage ranging from −0.0001 to +0.0001 V was used to collect data for a period of 10 s. For the calculation of the sheet resistance, the thickness and shape of the films were considered.

(3) $$R_{s}=\frac{\pi }{\ln\left(2\right)}\frac{∆V}{I}=4.53236\frac{∆V}{I}$$

where *V* is the measured voltage, and *I* represents the current applied. The resistivity is calculated as follows:

(4) $$\rho =R_{s}\;\times \;t$$

where *t* is the measured thickness.

Finally, electrical conductivity is calculated using the equation below:

(5) $$\sigma =\frac{1}{\rho }$$

#### 2.2.10. Photoluminescence (PL) Studies

The optical properties of the films were examined using the PL technique. A 375 nm CW laser (CNI, MDL-III-375) was utilized to stimulate the specimens. A laser beam with a 45° focused beam was directed toward the film sample surface, and perpendicular PL spectra were obtained using an Ocean Optics HR4000 (FL, USA) spectrometer. The color purity was calculated from the luminescence spectrum using this equation:

(6) $$Color\;purity=\frac{\sqrt{{\left(x_{s}-x_{i}\right)}^{2}+{\left(y_{s}-y_{i}\right)}^{2}}}{\sqrt{{\left(x_{d}-x_{i}\right)}^{2}+{\left(y_{d}-y_{i}\right)}^{2}}}\times \;100$$

where $\left(x_{s}-y_{s}\right)$ are the coordinates of a sample point, $\left(x_{d}-y_{d}\right)$ are the coordinates of the dominant wavelength, and $\left(x_{i}-y_{i}\right)$ are the coordinates of the illuminated point (0.333, 0.333).

## 3. Results

### 3.1. Chemical Composition, Structural Characterization, Physical, Mechanical and Optical Properties of Film Samples

#### 3.1.1. Fourier Transform Infrared (FTIR) Spectroscopy

**Figure 1:**
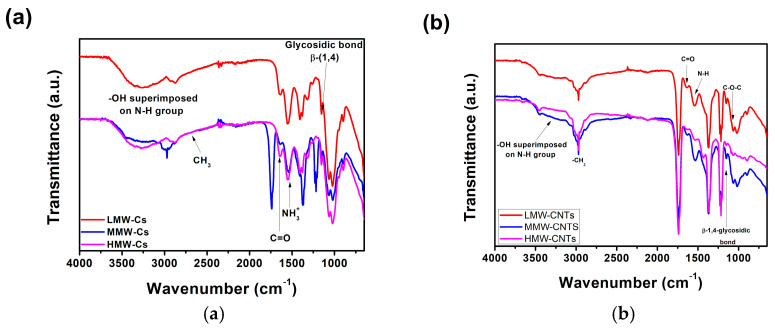
FTIR spectra of (**a**) pure chitosan films and (**b**) Cs/CNTs composite films.

The FTIR spectroscopy proved to be adequate for analyzing the functional groups of both pure Cs and Cs/CNTs films samples. Figure 1a depicts the FTIR peaks of films produced by pure Cs (LMW-Cs, MMW-Cs, and HMW-Cs). The spectra exhibit a broad-band spectrum ranging from the 3600–3200 cm^−1^, which corresponds to the stretching vibration of the -OH group. A broader superimposition is observed in the -NH_2_ and -OH regions because of the inter-hydrogen bonds of the carbohydrate ring. The bands located between 2869 and 2924 cm^−1^ are identified as antisymmetric and symmetric stretching vibrations of methyl (-CH_3_). The peaks at 1648 and 1643 cm^−1^ are associated with the stretching vibration of C=O from acetyl units of amide I. The peak located at 1538 cm^−1^, which is associated with the N-H bending vibration in the amide group, suggests that the amino group in Cs is NH_3_^+^ instead of -NH_2_.

In consequence, the electrostatic interaction between Cs and oxygen-containing groups on carbon nanotubes may be disrupted. The intensity of the NH_3_^+^ peak in chitosan film samples was significantly reduced, which may be attributed to the formation of hydrogen bonds between the hydroxyl groups of the solvent and the charged groups of Cs. The peak at 1378 cm^−1^ is due to a symmetrical angular deformation of -CH_3_ within the Cs molecule. The peaks of the β-1,4-glycosidic bond vibration and the C-O-C stretching vibration were located at 1151 cm^−1^ and 1094 cm^−1^, respectively. Furthermore, the MMW-Cs film displayed the more intense bands attributed to NH_3_^+^ groups, CH_3_ symmetric angular deformations, and the β-1,4-glycosidic bond. Otherwise, the bands attributed to NH_2_-OH, -CH_3_ antisymmetric deformations, and C=O from amide I vibrations are less intense in both LMW-Cs and HMW-Cs film samples [17,19].

Chitosan–CNTs film samples’ (LMW-Cs/CNTs, MMW-Cs/CNTs, and HMW-Cs/CNTs) FTIR spectra are depicted in Figure 1b. We observed that CNTs influence the vibrational groups of Cs. The band ranged from 3200 to 3600 cm^−1^, which is typical of -OH group, and appears less intense in comparison with the pure Cs film samples. The band attributed to symmetric and antisymmetric stretching vibrations of the -CH3 group was shifted to a high wavenumber due to the embedding CNTs. The peak associated with the stretching vibrations of C=O shifted to a low wavenumber (1630 cm^−1^). This indicates that Cs interact with the surface of CNTs, corroborating the Cs wrapping along the CNTs. The N-H bending vibration of the protonated amide group was shifted to a lower wavenumber (1532 cm^−1^), indicating that Cs positively interact with oxygen groups or defect sites on the CNT surface. The β-1,4-glycosidic bond of Cs shifted to a lower wavenumber (1148 cm^−1^), and the C-O-C stretching vibration shifted to a lower wavenumber, ranging from 1148 to 1063 cm^−1^. The LMW-Cs/CNTs film sample displayed the most intense bands in the FTIR spectrum, and these could be influenced by the long chain of Cs-wrapped on the CNTs [16].

Peaks at 3445, 1220, 1065, and 1603 cm^−1^ correspond to stretching vibrations from OH, C-O, C-O-C, and C-OH, respectively. The quinone-type units located along the sides of the CNTs appear as a sharp peak at 1638 cm^−1^ in the spectrum. CH_3_ vibrations were located within the range 3000–2800 cm^−1^. The centered band at 2999 cm^−1^ is attributed to the stretching of *sp*^2^-CH in benzene rings. Carbon nanotubes exhibit vibrations associated with carbon skeletal stretching in the range of 1580–1400 cm^−1^. It is possible that a low-intensity band, centered at 1637 cm^−1^, could be related to the conjugation of -C=C- with a carbonyl group. The occurrence of the band centered at 1092 cm^−1^ could be attributed to the shift in C-O-C absorption to lower frequencies, which results from the interaction between the -OH from Cs and the rings of CNTs [17]. We hypothesized that, as the Cs molecular weight increased, more Cs molecules could interact with the surface of CNTs. This would result in a decrease in the intensity of the characteristic vibrations of the CNTs, and thus an increase in the Cs vibrations in the FTIR spectra. The typical vibrations of carbon nanotubes at 1604 cm^−1^, which are attributed to C=C asymmetric stretching, are not detected in the FTIR spectra [20]. This demonstrates that the Cs chains are successfully wrapped around the surface of CNTs. A second-derivative analysis is depicted in the Supplementary Information (Figure S2).

CNTs may possess oxygen functionalities that may be categorized as structural defects, such as edges and basal planes responsible for lattice vacancies. The incorporation of CNTs within Cs exerts an influence on hydrogen bonds, thereby facilitating interactions throughout the entire system. This further reduces the availability of protonated groups in Cs, aiding the buildup of wrapped structures.

The bands corresponding to amine groups in Cs seem to be more intense in MMW-Cs and HMW-Cs composite film samples. The observations suggest the possibility of interaction between positively charged carbon nanotubes and oxygenated groups. As per previous reports, extensive hydrogen bonding has been observed in the films of LMW-Cs/CNTs [21].

#### 3.1.2. Thickness 

The thickness of Cs and Cs/CNTs film samples is presented in Table 1. The thickness values of Cs films, namely LMW-Cs, MMW-Cs, and HMW-Cs, were recorded as 66 µm, 54 µm, and 62 µm, respectively. Our findings are in agreement with the average thickness values of pure Cs reported by Saputra et al. [22]. The Cs/CNTs film samples displayed average thicknesses of 84, 66, and 54 µm for LMW-Cs/CNTs, MMW-Cs/CNTs, and HMW-Cs/CNTs film samples, respectively. By adding carbon nanotubes to Cs, a thicker film was produced, suggesting that CNTs strongly interact with the chains of Cs approaching crystalline structures by reducing the interplanar distance. Aryaei et al. reported an average thickness of 60 µm of dried uniform thin films made of Cs and multiwalled carbon nanotubes [23]. LMW-wrapped chitosan along the CNTs surface could assemble outside the polymeric matrix, resulting in thinner films, which is in good agreement with Rodrigues et al. [24].

#### 3.1.3. Ion Exchange Capacity (IEC)

IEC is a parameter that allows us to quantify the ability of functional groups to bind protons in a liquid solution and evaluate the proton conductivity within the film. The parameter provides a correlation between the quantity of interchangeable ionic groups that contribute to water absorption. In pure Cs film samples, the ionic groups acting as ion exchangers comprise -NH_3_^+^, and an increase in the quantity of protonated groups results in an increase in the IEC values. The Cs/CNTs film samples displayed an increased %DS, which in turn resulted in higher IEC values in comparison to the pure Cs samples. Biopolymer films display high water absorption capacity due to the high availability of -OH and NH_3_^+^ groups within Cs chains. Our findings suggest that the enhanced IEC is due to the chitosan wrapped along CNTs, which expose the charged groups suitable for ionic conductivity. An increased IEC value is accompanied by a rise in water absorption. According to Nhung et al., hydrophilic polar groups in Cs lead to an increase in the swelling ratio and IEC values, which in turn contributes to the film’s water absorption ability. The transfer of ions is dependent on the absorption of water, and improving water uptake may result in enhanced ionic conductivity [25].

The transport charge is improved by CNTs due to their unique electronic properties. The highest IEC values reported in this study were obtained by the Cs/CNTs film samples, which can be attributed to the Cs-wrapped along CNTs and its efficacy in enhancing charge transport. Table 2 shows the IEC values of both Cs and Cs/CNTs film samples.

#### 3.1.4. Degree of Swelling and Solubility

Our results agree with the typical values of three-dimensional polymeric networks suitable for swelling without altering their structure for up to 48 h, after which the absorption equilibrium is reached, previously reported by Lewandowska et al. [26].

We previously demonstrated that pure MMW-Cs film has a maximum swelling percentage of 798%. After incorporating CNTs into MMWS chitosan, the %DS value decreased as the Cs molecular weight increased (Table 2). This could be due to hydrophilic Cs along with unbound polar groups that interact with water molecules. As reported by other authors, the hydrophilicity of the polymeric matrix could be reduced due to the addition of CNTs [27].

Cs/CNTs film samples exhibit significant decreases in the degree of swelling values of 36% and 11% for both MMW-Cs/CNTs and HMW-Cs/CNTs films, respectively. This tendency may be related to the build-up of a more condensed structure that may lead to a reduction in swelling capabilities of the biopolymer films. The incorporation of CNTs led to a decrease in hydrophilic behavior, which reduced the number of oxygen-containing groups and thus hindered the availability of polar groups. This resulted in lower swelling capabilities of the films. The FTIR spectrum of Cs/CNTs film samples revealed a decreased intensity of the OH band, suggesting an interaction between positive Cs and oxygen-containing groups of CNTs.

A maximum degree of swelling has been attributed to the availability of amine groups interacting with the hydrophilic groups of water. According to the FTIR analysis, the superimposed OH-NH_2_ band is more intense in the MMW-Cs film than in the LMW-Cs film sample and LMW-Cs films. The percentage of SD observed in films containing CNTs increases as chitosan molecular weight increases. For all three molecular weights of chitosan with CNTs, the intensity of the superimposed OH-NH_2_ band was higher for the LMW-Cs/CNTs sample films. The decrease in SD can be attributed to the presence of CNTs, which confer higher negative charges, which in turn cause higher repulsion of water molecules [28].

#### 3.1.5. XRD Analysis

**Figure 2:**
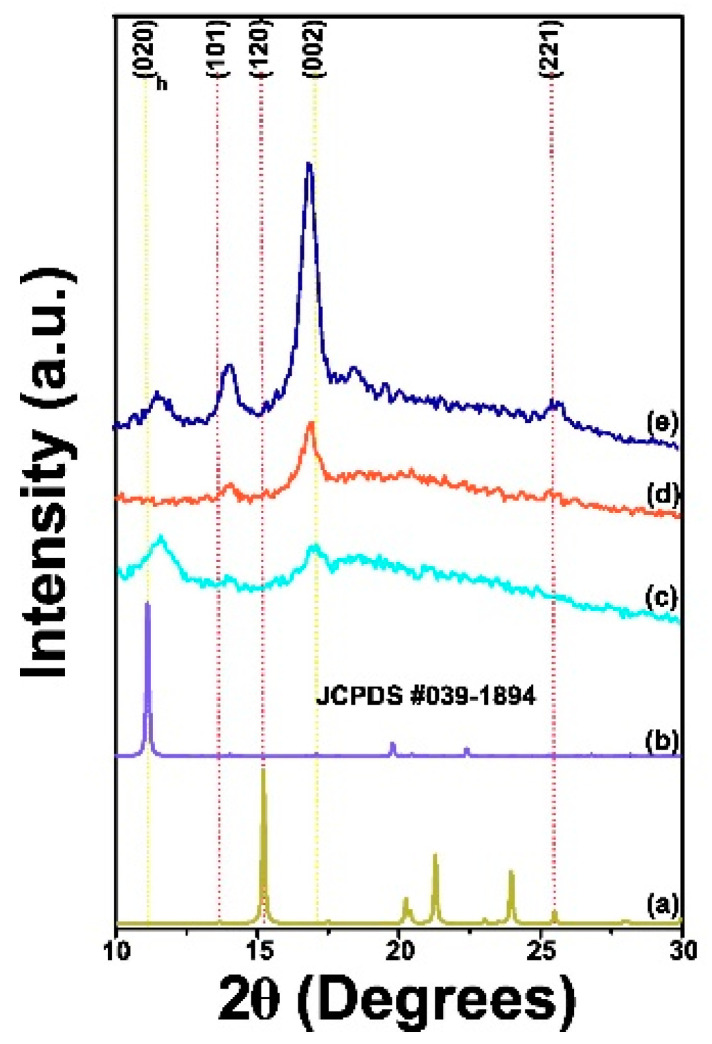
XRD patterns of (a) chitosan crystal form I, (b) chitosan crystal form II, (c) LMW-Cs, film, (d) MMW-Cs film, and (e) HMW-Cs film.

Chitosan can present two orthorhombic crystal forms. The form I crystal structure exhibits a strong reflection centered at 2θ = 11.4°, while the form II crystal exhibits a prominent reflection at 2θ = 20.1° for the (102) reflection as per the reference code JCPDS #39-1894, as depicted in Figure 2a,b.

**Figure 3:**
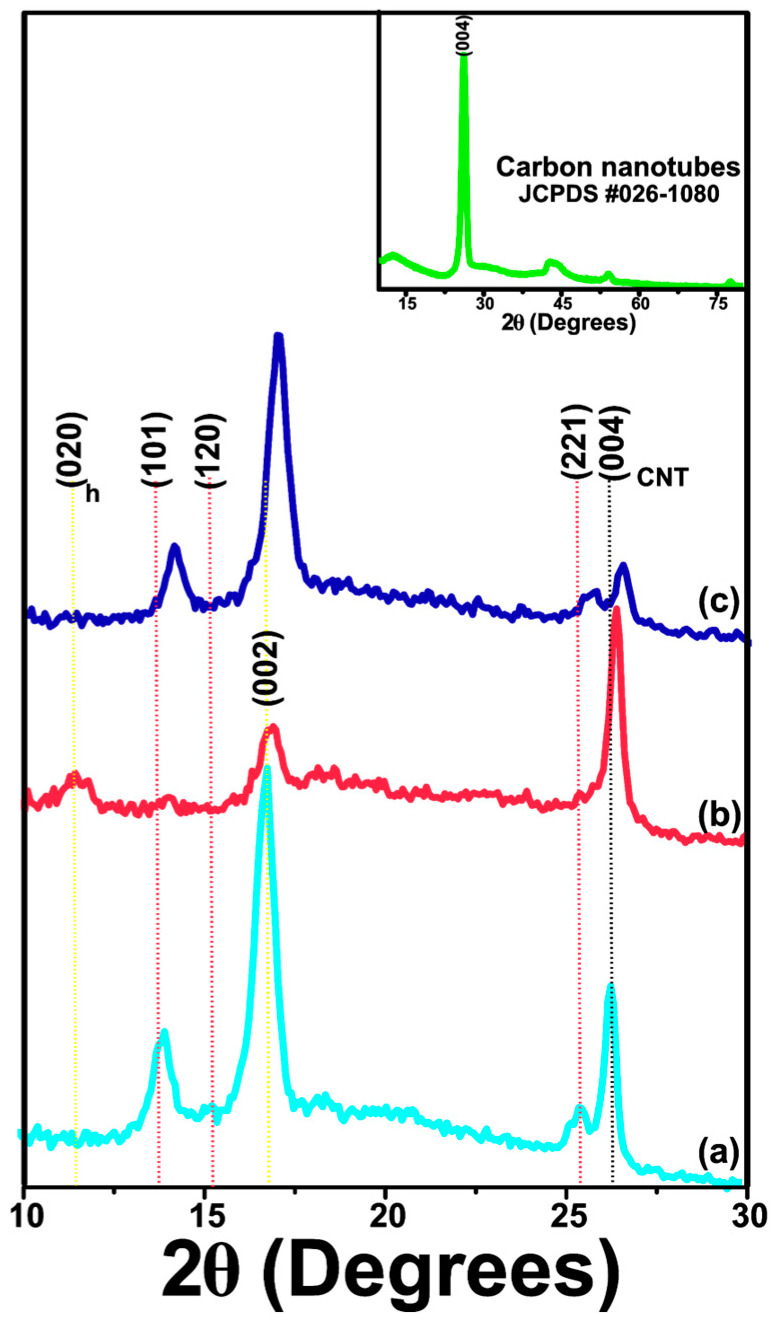
XRD patterns of (a) LMW-Cs/CNTs film, (b) MMW-Cs/CNTs film, and (c) HMW-Cs/CNTs film. The inset exhibits the XRD pattern of carbon nanotubes.

In Figure 2c, the diffractogram of pure LMW-Cs film sample demonstrates reflections associated with crystalline and amorphous regions, centered at 2θ = 11.5°, 14.09°, 16.93°, 18.37°, and 25.51°. The XRD pattern of pure MMW-Cs film is shown in Figure 3b and demonstrates reflections associated with the crystalline and amorphous regions centered at 2θ = 14.05° and 16.87°. Figure 3c depicts the XRD pattern of a pure HMW-Cs film, exhibiting both crystalline and amorphous regions, centered at 2θ = 11.45°, 14.04°, and 16.87°. The appearance of a peak associated with the (020)_h_ plane, indicates that the formation of crystal form I is observed in films produced with low- and high-molecular-weight chitosan, whereas films produced with medium-molecular-weight chitosan show crystalline structure form II. In all pure chitosan films, the (002) plane shifted towards lower angles and is accompanied by an increase in its intensity as a function of molecular weight.

After the incorporation of carbon nanotubes (JCPDS 00-026-1080), we observed that LMW-Cs/CNTs’ film diffraction pattern slightly shifts towards higher angles (2θ = 14.08°, 16.87°, 19.74°, and 25.63°), as depicted in Figure 3a. Interestingly, the reflection associated with the chitosan crystal form I completely disappears, suggesting that, by adding carbon nanotubes, a structural modification occurs. The signal ascribed to the (004) plane of carbon nanotubes at 2θ = 26.38° confirmed that carbon nanotubes were effectively embedded into the chitosan framework.

The XRD pattern of MMW-Cs/CNTs film is shown in Figure 3b. After the CNTs incorporation, we observed that the diffraction pattern depicts two signals at 2θ = 11.65° and 16.98°. The signal attributed to the (004) plane of carbon nanotubes was observed at 2θ = 26.59°. Figure 3c displays the XRD pattern of HMW-Cs/CNTs film signals at 2θ = 14.02°, 16.92°, 22.17°, and 25.73°. The signal attributed to the (004) plane of carbon nanotubes was observed at 2θ = 26.45°. The enlarged (004) plane is shown in the Supplementary Information (Figure S3).

All XRD patterns of Cs/CNTs films samples displayed a shift towards higher angle values. This is attributed to both inter- and intra-layer strain and may be associated with the interaction between chitosan and CNTs. The intercalation between CNTs and amine groups may influence the crystalline structure of Cs, as evidenced by previous reports [29]. From our findings, we report that after the incorporation of CNTs resulted in a rise in the intensity of crystalline reflections of Cs.

The film-forming process may be responsible for the significant variances observed in the XRD patterns compared to other reports in the literature [30,31]. The peak at 2θ = 10–13°, belonging to chitosan crystal I form, suggests that hydrated chitosan is slightly preferred over both LMW-Cs and HMW-Cs films in the XRD pattern, whereas it is not found in films containing CNTs (LMW-Cs/CNTs; HMW-Cs/CNTs).

The reflection (020)_h_ was no longer observed in pure MMW-Cs film sample; however, it was further observed after the incorporation of CNTs. We conjecture that embedded CNTs in pure MMW-Cs exhibit a more hydrated form due to the wrapped chains along the CNT surface. The diffraction peaks with the highest intensity are observed between 2θ ~11–13 and 15–17° and are assigned to the (020)_h_ and (120) crystalline planes, respectively, while the secondary peaks are evidence for a mixture of both crystals form I and II. The absence of the (120) plane in chitosan anhydrous crystal form II is related to the O3···O5 bond of the hydrogen bond, which contributes to the stabilization of a twofold helical conformation by decreasing the deacetylation degree, according to Facchinatto et al. [32]. The possible hydrogen bond between C=O···HNC and C=O···HOC can be reduced, resulting in a diffraction pattern with low crystallinity.

The crystalline structure of chitosan also can be affected by the high penetration of water molecules, which reduces the average crystallite size and causes an expansion of the structure along the axis. This occurs because there is no inter-sheet hydrogen bridge between the C(O)···HOC. Furthermore, the hydrated form of chitosan preserves the N2···O6 bond, thereby facilitating an inter-sheet arrangement. The structural arrangement of chitosan forms I and II is determined by the hydrogen bridge bond N2···O6 formed between adjacent (hydrated) sheets along the b-axis, whereas in anhydrous chitosan, adjacent sheets form hydrogen bridge bonds along the a-axis between N2···O6. It is inferred that the crystalline arrangement changes from a hydrated to an anhydride form is due to the incorporation of carbon nanotubes. Since the chitosan is wrapped onto the carbon nanotubes, the hydrogen bonds between adjacent polymer sheets are reduced [32]. A hydrated crystalline structure of the MMW-Cs/CNTs film favors the formation of hydrogen bonds.

The decrease in d (002) spacing in the Cs/CNT network due to strain in both inter- and intra-layer directions leads to a rearrangement of the hydrogen bonds between chitosan and CNTs, suggesting that carbon nanotubes influence chitosan structural properties. XRD analyses demonstrate that CNTs have been intercalated within the chitosan structure, thus widening the basal spacing d (002). The preparation of chitosan-based films resulted in a crystalline disruption, which was reflected in a shift in the peaks attributed to both hydrated and anhydrous forms. This change has a significant impact on the crystallinity of the films examined in the current research.

Understanding the structural properties of the chitosan-based films, which directly influence the mechanical, chemical, and biological behavior of the material, is imperative for determining their efficacy in biomedical applications. Crucial insights into the internal arrangement of chitosan, which influence its strength, flexibility, resistivity, and biocompatibility, are provided by the crystallinity index, crystallite size, microstrain, and dislocation density. The crystallinity index (CrI) is a measure of the degree of order in a polymer, which has the potential to influence its stability. The crystallite size is a measure of the size of the crystalline domains, which influences the toughness of the polymer and its response to mechanical stress, which is crucial for biomedical devices. Microstrain provides information about internal stresses and defects within the polymer structure, which can influence its mechanical properties and potential for failure under stress. In addition, the dislocation density reflects the occurrence of defects or dislocations within the polymer matrix, which can play a role in the toughness and resistance to fracture of the material. By evaluating these parameters, we gain a comprehensive understanding of the structural integrity of polymer-based films. This allows us to optimize its properties for efficient use in bioengineer applications, such as drug delivery systems, tissue scaffolds, and medical implants.

From our findings, we report that CrI values (Table 3) of pure chitosan-based films tend to increase as a function of the molecular weight. The correlation between the structural features of Cs and its CrI, a measurement of the degree of order in the polymer structure, is evident. Since the observed XRD signals were broad, it was necessary to analyze them using the deconvoluting method. According to Barbosa et al., the chitosan molecular weight influences the crystallinity of LMW-Cs, MMW-Cs, and HMW-Cs film samples [33]. The comparison between crystalline and amorphous regions is critical for determining the functional features of the sample films, which has significant implications for the applications of Cs-based films. Depending on the calculation methodology employed, the reported CrI values may show significant variation. It is crucial to consider the method used carefully to obtain an accurate characterization of the films.

CrI measurements were conducted according Podgorbunskikh et al. [34], using the ratio between the intensities of the crystalline and amorphous regions at 2θ = 19–20° and 2θ = 12–16°, respectively, with $\left(CrI=S_{cryst}/S_{total}\right)$. However, the effectiveness of the approach is significantly decreased when a mixture of anhydrous and hydrated polymorphs is observed. For more details, see Supplementary Information (Figure S4). Even though chitosan is a semicrystalline material, the processing and filler nature have the potential to modify the crystallinity of the material and, consequently, properties such as mechanical, optical, and electrical. It is noteworthy that all the films analyzed in our research exhibited enhanced properties; however, it was MMW-Cs/CNTs films that exhibited the highest electrical conductivity value and enhanced mechanical properties. The diffraction patterns of anhydrous and hydrated forms of Cs previously reported are quite different from those reported in the present study [30,31].

From the XRD data, it is possible to calculate crystallite size, dislocation density, and strain by using the Williamson–Hall (W-H) model, which is relevant for the comprehensive understanding of the structural modification due to the CNTs incorporation.

Using W-H plot, the particle size and strain of composite films produced with low-, medium-, and high-molecular-weight chitosan, considering high-intensity peaks (020)_h_, (101), (120), and (002) of chitosan and high-intensity peak (004) belonging to CNTs, are calculated. The crystallite size (D), dislocation density, and microstrain (ε) of all the composite films for mentioned peaks are given in Table 4.

(7) $$\beta Cos\theta =\varepsilon \left(4Sin\theta \right)+\frac{K\lambda }{D}$$

Full width at half maximum (FWHM) values, denoted by *β*, are measured for each peak within the X-ray diffraction pattern. The respective strains, *ε*, are also considered. The half-angle values, *θ*, of the peaks are used with the form factor, *K*, and the wavelength of the X-rays, *λ*, to calculate the crystallite size, *D*.

**Figure 4:**
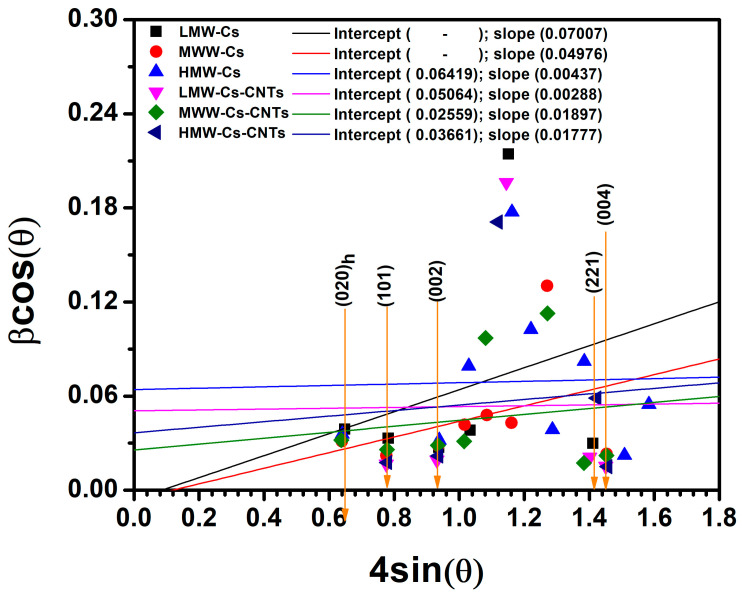
Linear fit of the W-H model obtained from the XRD patterns of the film samples of both pure chitosan and chitosan–CNTs.

Figure 4 depicts the W-H plots for all film samples, distinguished by respective colors. In these plots, the most important parameters are the slope and the intercept with the “Y” axes of their linear fits. The W-H analysis is an integral breadth method where both size and strain-induced broadening are considered in the deconvolution of the peak versus 2θ. From our findings, we reported that the chitosan structure is highly influenced by CNTs incorporation.

The crystallite size indicates the degree of crystallization and structural quality of the film samples. A larger size of crystallite is associated with fewer defects and a higher degree of crystallinity. The MMW-Cs/CNTs film sample suggests that they display a more ordered structure, which is attributed to the carbon nanotubes incorporated into the crystal lattice of chitosan. Microstrain values indicate that the molecular weight of chitosan plays a significant role in the concentration of defects. Our findings suggest that both the LMW-Cs/CNTs and MMW-Cs/CNTs film samples exhibited a low defect density, which may be attributed to dislocations or small grain boundaries. Interestingly, as observed in the SEM images, the MMW-Cs/CNTs film sample may contain significant defects that cause distortion in the crystal structure of chitosan due to CNTs but enhanced resistivity properties, maybe due to the tube–tube interaction due to the structural arrangement. Even when the microstrain is increased in MMW-Cs/CNTs film samples, the crystallite size is still high. The dislocation density values are useful in understanding the crystalline quality of the film samples and provide an insight into how the materials could behave mechanically and electrically for the film’s applications proposed in this research. After considering all parameters analyzed from the W-H plot, it appears that the LMW-Cs/CNTs film sample has a more crystalline structure with the lowest dislocation density. This indicates a more structured structure with less distortion. Although having the largest crystallite size, the MMW-Cs/CNTs film sample has the highest microstrain and dislocation density. This could suggest that, even with a very large crystallite size, the material could be highly defective or disordered, suggesting a less crystalline film. However, there are two interesting points to consider, such as the molecular weight of the chitosan and carbon nanotubes involved in the wrapping of the chitosan along the CNTs. As a result of shorter chains that indicate less entanglement, the LMW-Cs/CNTs film samples may exhibit reduced crystallinity and poor mechanical properties, since crystallizing of chitosan is influenced by its molecular weight.

Poor CNT dispersion can result in local stress concentration or defect formation, and their orientation can have an impact on the mechanical and electrical properties of the film. Furthermore, randomly oriented CNTs can result in inadequate mechanical and electrical performance. MMW-Cs/CNTs film samples could provide an optimal balance between chain length and entanglement for more effective crystallization influenced by CNT dispersion within the polymer. This results in a larger crystallite size, lower microstrain and dislocation density, and improved mechanical and electrical performance.

The W-H plot helps us to distinguish the effects of crystallite size, dislocations density, and microstrain. Larger crystallites typically are associated with a higher degree of crystallinity as the crystal domains are well-ordered. Microstrain, however, refers to internal strain or defects in the crystal lattice that cause distortion of the diffraction peaks. Since high microstrain often correlates with low crystallinity or high defect concentration, the relationship between crystallinity and microstrain could indicate that the crystallite size is larger than the microstrain. Dislocations can cause local distortions in the crystal lattice. High dislocation densities typically indicate a more deformed or defective material, as they may cause local distortions in lattice spacing (d), which may lead to modifications to the microstrain and broader peaks in the XRD pattern [35,36,37]. This is because it corresponds to less ordered atomic structures due to the accumulation of dislocations, resulting in a decrease in overall crystallinity.

Chitosan chains wrapping carbon nanotubes have the potential to influence structural properties of the chitosan-based films. The wrapping can cause local distortions in the polymer chains arrangement, which can lead to microstrain in the polymer matrix. This is recognized in the XRD pattern by the broadening of diffraction peaks and shifting of peak positions due to lattice strain. Since carbon nanotubes can act as a site for dislocation [38,39], the wrapping may restrict the movement of the polymer chains and introduce disorder into the films. Wamuo et al. [40] reported that crystallization behavior of polymer chains can significantly reduce the mobility and further crystalline growth, which, in our research, may impede the mobility around the CNT surface, thereby preventing the formation of extensive crystalline domains, resulting in disordered and smaller crystallites. The overall performance of the chitosan-based film samples investigated in this study is due to the favorable interaction between the polymer chains and the tube–tube interaction favored by the wrapping.

#### 3.1.6. X-Ray Photoelectron Spectroscopy (XPS)

**Figure 5:**
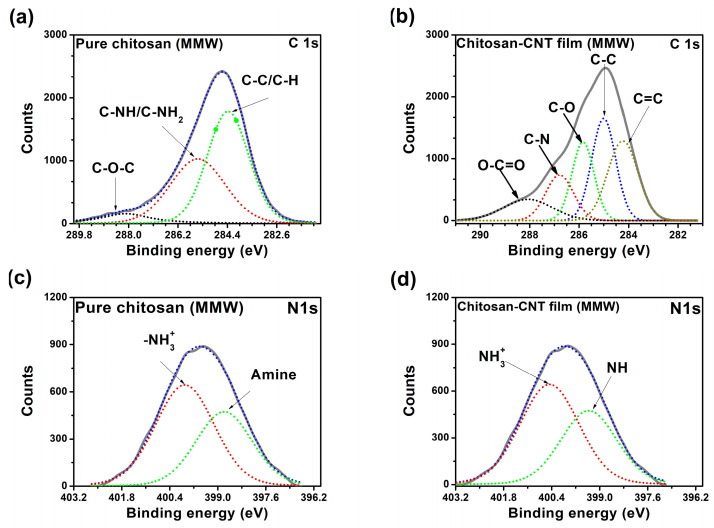
XPS core level C 1s from (**a**) pure MMW-Cs film (red-colored line indicates the C-N feature; green-colored line indicates the C-C/C-H feature), (**b**) MMW-Cs-CNT film (brown, blue, green, red and black-colored lines line indicate the C=C, C-C, C-O, C-N, and C=O features, respectively. The N1s core level from (**c**) pure MMW-Cs film (red-colored line indicates the -NH_3_^+^ feature; green-colored line indicates the amine feature) and (**d**) MMW-Cs-CNT film (red-color line indicates the -NH_3_^+^ feature and green-colored line indicates the -NH feature) is shown.

The XPS measurements allow for the identification of the core levels of O 1s, C 1s, and N 1s in the MMW-Cs/CNTs film. We obtained the photoelectron-binding energies (BEs) from their corresponding peak positions in the XPS spectrum (for more details, refer to Figure S5). For spectral calibration, the BE of the C 1s line at 284.5 eV was used as the reference. Figure 5c,d show the XPS N 1s core-level spectra of both pure MMW-Cs and MMW-Cs/CNTs sample films. The C-C peak of chitosan is slightly shifted to lower BE values when compared to the MMW-Cs/CNTs C 1s binding energy, as depicted in Figure 5a. The interaction between chitosan and carbon nanotubes could be confirmed by the nonexistence of the π-π* shake-up feature, typical of aromatic structures. The absence of this feature could indicate that the wrapping of chitosan disrupts the π-conjugated system of the CNTs, possibly due to steric hindrance or electronic interactions that alter the electronic environment around the carbon atoms. Furthermore, it can lead to a chemical bonding or physical interaction that modifies the electronic properties of the CNTs by forming strong hydrogen bonding or electrostatic interactions, which thereby suppress the π-π transitions due to new electronic states that could mask or eliminate the feature shake-up. According to Yang et al., the internal features of the N 1s core-level at 399.4, 400.5, and 401.7 eV are associated with the amine, amide, and protonated amine groups [41], respectively. Our results suggest that N 1s spectra of MMW-Cs/CNTs film has two inner components: the peak at 399.32 eV is associated with the amine group, and the peak at 400.38 eV is ascribed to the protonated -NH_3_^+^ group (Table 5).

#### 3.1.7. Mechanical Properties

When evaluating the mechanical performance of films, consider parameters such as tensile strength (TS), elongation-at-break (EB), Young’s modulus, and modulus of toughness. These parameters have a direct correlation with the structural cohesivity of the test film samples, indicating their resistance to breaking under tension and fracture strain subsequent damage.

It has been reported that achieving good dispersion and a homogenous film is a technical defiance when studying the mechanical properties of Cs/CNTs films. This is due to the intrinsic van der Waals forces, which induce the aggregation of CNTs, which affects the mechanical performance of the films [42,43]. In previous research, we reported the mechanical properties of pure LMW-Cs, MMW-Cs, and HMW-Cs films [6]. These values were used as a reference for Cs/CNTs films mechanical performance, presented in Table 6. The results revealed that adding CNTs had a significant influence on the mechanical properties of all film samples. The LMW-Cs/CNTs film displayed detrimental mechanical performance, which could be attributed to the fact that molecular weight Cs plays a key role in the mechanical performance of the films.

The film produced with MMW-Cs/CNTs demonstrated the most significant enhancement in mechanical performance in comparison to the pure Cs film. The values were enhanced by 143%, 152%, 116%, and 217%, respectively, for TS, EB, Young modulus, and toughness modulus (Table 6). This increase demonstrates a good dispersion and attractive interaction between the pure MMW-Cs and CNTs, which could be attributed to the wrapped chitosan along them and thus to the increased toughness modulus values since the MMW-Cs/CNTs films increased to 2.55 MJ m^−3^, which implies that these films can absorb this amount of energy per volume before rupture.

It has been reported that hydrated Cs films samples yield rupture values of close to 10–30 MJ m^−3^, whereas biopolymers carbon nanotube network films exhibit rupture values of up to 45 MJ m^−3^. The rupture values are determined by the nature of the materials suitable for releasing stress in films containing carbon nanotubes. The decrease in mechanical performance is attributable to chitosan-wrapped along carbon nanotubes [42,43,44,45]. The distribution of filler content and orientation have the potential to influence the mechanical properties of polymeric films, as demonstrated by previous research [46]. Rodríguez et al. reported that an inadequate dispersion of CNTs resulted in a factor that influence the mechanical performance because it leads to the formation of nanocracks [24]. As dispersions undergo ultra-homogenization, polymer molecules are subjected to mechanical stress as a result of the concurrent shear, turbulence, impacts, and cavitation forces [47].

The study conducted by Zhang et al. reported the enhancement of the mechanical performance of Cs through the combined use of montmorillonite and CNTs. The authors proposed that a synergistic process could be initiated by the positively charged polymeric chains that absorb onto the CNTs surface, leading to wrapping along CNTs [48].

Based on our FTIR findings, it has been observed that as the intensity of the amine band decreases, the elongation-at-break also increases, if it is accompanied by a good TS value, the film samples can be of superior quality.

#### 3.1.8. Scanning Electron Microscopy (SEM) Analysis

**Figure 6:**
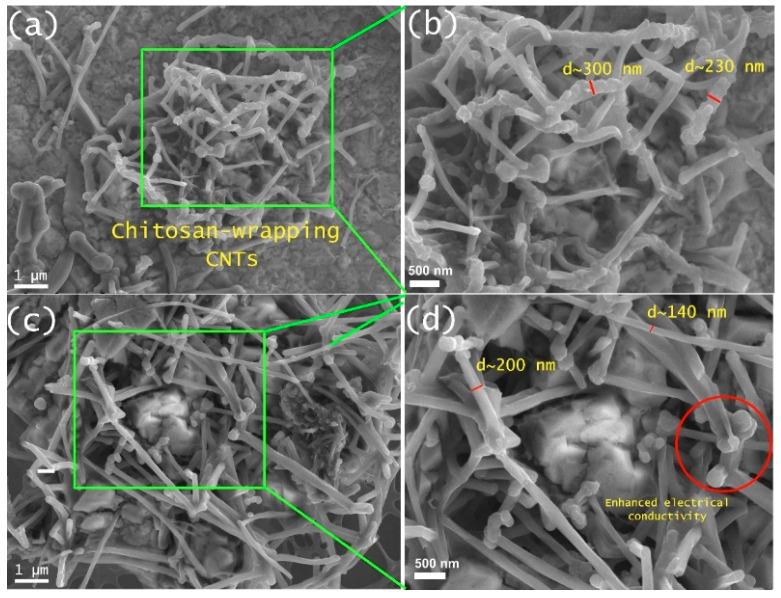
SEM of (**a**,**c**) MMW-Cs Cs/CNTs film and (**b**,**d**) zoomed-in section of MMW-Cs Cs/CNTs film.

Figure 6a–d display SEM images, and a zoomed-in section of the Cs/CNTs film is shown. The pristine carbon nanotube diameter ranges between 6 and 13 nm, and the increased diameter is attributed to the wrapping of chitosan onto the CNTs surface [49]. The complete coverage of the carbon nanotube surface with Cs bumps is attributed to its high polar nature, which results in enhanced wrapping capabilities [50]. It has been observed that the NH_3_^+^ groups in Cs contribute to the wrapping of CNTs, acting as active polar groups within their vicinity and being able to interact with the CNTs surface through van der Waals interactions. The favored free energy leads to spontaneous wrapping of CNTs, taking, and helical conformation, resulting in the formation of a thin layer on CNTs surface [51].

Figure 6 demonstrates the influence of CNTs in the pure MMW-Cs film. The coverage of chitosan along the carbon nanotubes is clearly visible, exhibiting tube-like structures with diameters ranging between 140 and 300 nm. This has important implications for the understanding of the mechanical performance of the film samples. TS and EB values obtained from mechanical analysis may be attributed to the protrusions of Cs that were formed on the surface of CNTs and eventually wrapped around them, as depicted in SEM images.

#### 3.1.9. Four-Probe Measurements

The resistivity of all sample films was measured using a Keithley Model 4200-SCS system. All measurements were performed using equidistant tip configuration (0.040 in). The data were collected for 10 s with a gate voltage between −0.001 and +0.001 V. The resistivity (ρ) was calculated from the characteristic *I-V* curves. Sheet resistance (Ω/Υ) was calculated according to Equation (3).

Table 7 displays the electrical properties of pure Cs and Cs/CNTs film samples. An average conductivity of 5.1 × 10^−8^ S cm^−1^ was calculated in pure LMW-Cs film, indicating a slight increase from 5.95 × 10^−8^ and 5.16 × 10^−8^ S cm^−1^ for MMW-Cs and HMW-Cs film samples, respectively. Cs/CNTs film samples yielded electrical conductivity values of 0.0048, 0.0156, and 0.0132 S cm^−1^ for LMW-Cs/CNTs, MMW-Cs/CNTs, and HMW-Cs/CNTs film samples, respectively. Our findings allow us to conjecture that the formation of an interconnected Cs/CNTs network has the potential to enhance the electrical properties of Cs film samples. Falamarzpour et al. reported that the incorporation of a small quantity of CNTs enhanced the electrical conductivity of a chitosan nanocomposite. The improved electrical conductivity was attributed to a synergistic interaction between CNTs and Cs, along with a good dispersion and overlapped structure [10].

The significantly improved electrical properties of Cs/CNTs film samples are assumed to be due to the interactions between the tubes and their dispersion within chitosan, ensuring the efficient transfer of electrical charge. The sheet resistance (R_s_) is used to characterize semiconducting and conducting materials. The measurement of the lateral resistance per square area of a film with uniform thickness is used to evaluate the capacity of an electrical charge to travel within the film. From our findings, we report that an increase in the chitosan molecular weight decreases the R_s_ value. The addition of CNTs resulted in lower R_s_ values for LMW-Cs/CNTs (209.9 mΩ/Υ), MMW-Cs/CNTs (16.7 mΩ/Υ), and HMW-Cs/CNTs (174.8 mΩ/Υ) film samples by six orders of magnitude. According to Lau et al., the resistivity values of Cs/CNTs solutions are influenced by the weight percentage of CNTs present in the solution [52]. It has been demonstrated that the embedding of CNTs into chitosan results in enhanced electrical conductivity, thereby enabling films with low R_s_. A significant contribution has been made to studying conductive films that are reinforced with CNTs to enhance their electrical properties. For more details, refer to the Supplementary Information file (Figure S6). The incorporation of CNTs into Cs may serve as an alternative to the commonly studied fillers, such as silver nanowires, copper, conductive polymers, such as PEDOT, or polyaniline [53,54,55].

#### 3.1.10. Photoluminescence (PL) Analysis

For PL measurements, film samples were excited using a CW solid-state laser (λ_exc_ = 375 nm, E = 100 mJ), focused at 45° to the sample surface. PL emission was acquired perpendicular to the sample surface using a lens array. PL emission was focused onto a fiber bundle and guided to an OCEAN OPTICS HR4000 spectrometer coupled to a computer, where spectra were finally processed. Within the process of PL, a molecule absorbs a photon, leading to the excitation of its electrons to a higher electronic level [56,57,58].

**Figure 7:**
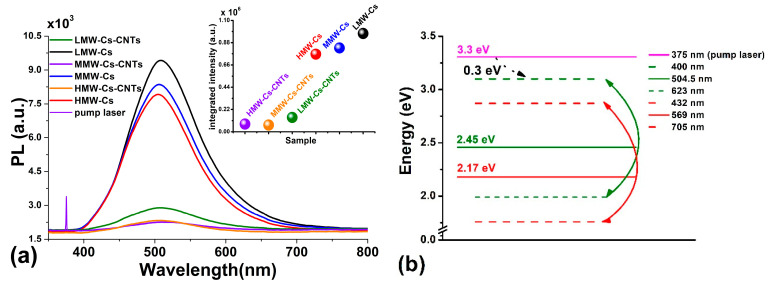
PL studies: (**a**) PL spectra of pure Cs and Cs/CNTs films from LMW-Cs to HMW-Cs. (**b**) Energy diagram.

Figure 7 shows the PL emission spectra of both Cs and Cs/CNTs film samples. For comparison, the pumping laser line is also presented in the figure. Chitosan film samples exhibit broadband luminescence in the visible spectral region ranging from 400 nm to 700 nm. The inset exhibits integrated intensities of the films. It is important to mention that no emissions were detected in either the NIR or IR spectral regions. The results of integrated intensity show that the PL intensities of Cs/CNTs films are quite lower than emission of pure Cs film samples. The results of spectra deconvoluting reveal that the spectral structures of both pure Cs and Cs/CNTs films are quite similar. The spectra are composed basically of two emission bands. For LMW-Cs and LMW-Cs/CNTs film samples (Figure 7a), these bands are centered around 504.5 nm (2.45 eV) and 567 nm (2.17 eV), respectively. This suggests that CNTs do not contribute to luminescence, most probably because Cs is wrapped around their surface, and the laser radiation mainly excites the Cs molecules. To explain the absorption–emission processes, an energy diagram (Figure 7b) was constructed using the pumping laser line, the peak positions, and the extremes of the two emission bands detected in PL spectra after deconvoluting.

Upon excitation with λ = 375 nm, electrons are pumped to the LE (3.3 eV) level. However, the luminescence onset is observed at 400 nm (3.0 eV); consequently, electrons should lose at least ~0.3 eV energy through non-radiative transitions before they begin to decay radiatively to the ground state. Furthermore, the fact that no emissions were detected in the IR region excludes radiative transitions between the observed luminescence bands. In the case that radiative transitions occur within the luminescence bands, the emissions would be in the infrared region (λ > 700 nm; E < 1.7 eV). Consequently, the observed emission of pure Cs films is structured by phonons. Electrons lose energy via phonons and finally decay radiatively to the ground state, conforming to the observed luminescent bands. According to FTIR, vibrations around 3700–3000 cm^−1^ possess energies of 0.3–0.4 eV.

The occurrence of macroscopic defects (e.g., defects, local states, holes, pores) in chitosan and carbon nanotubes may influence the PL transitions. The wrapping contributes to chitosan being attracted to the CNTs surface and shortens the bond distance, leading to energy transfer between nearby atoms leading to a non-radiative process. PL emission of chitosan is ascribed to the large quantity of oxygen atoms and the acetamide group. Finally, the electrons return to a lower energy level, and as a result, a photon is emitted. Oxygen atoms with high electron density serve as luminescent centers, thereby emitting light. Chitosan–CNTs film samples produced in this research exhibit green emission (504–510 nm), which suggests that the incorporation of CNTs provokes a wavelength shift towards lower values. It can be inferred that point defects appearing in CNTs are the source of green emission [6,59].

The parameters of the International Commission on Illumination (CIE), such as color coordinates (x, y), were calculated to know the color attributes for the Cs films and the Cs/CNTs. The Supplementary File (Figure S7) depicts the CIE chromaticity diagram of the films. The color coordinates traverse a brief range from red to blue when CNTs are incorporated into the Cs. The CIE-1931 coordinates of the Cs film samples shifted from green (LMW-Cs films) to green–blue (MMW and HMW chitosan films) as the molecular weight of the Cs increased. The calculated results presented in Table S2 indicate that the red color purity decreases with the addition of CNTs, which is in accordance with the color attributes L, a*, and b* depicted elsewhere in the Supplementary Information file (for more information, refer to Table S1). The energy diagram showed that by incorporating CNTs, a reduction in the energy bands was observed.

## 4. Conclusions

This study has demonstrated that chitosan molecular weight has a significant impact on the properties of biopolymer films. According to XRD measurements, reflections related to both crystal forms I and II were observed in both pure LMW-Cs and HMW-Cs film samples, whereas crystal forms I and II were observed in pure MMW-Cs film. Furthermore, by incorporating carbon nanotubes, a structural modification has occurred, as evidenced by the reflection (020). These findings suggest that the modifications in crystalline arrangement resulted in a more hydrated crystalline structure of MMW-Cs/CNTs film, which favors the formation of hydrogen bonds. This has significant implications for the improvement in TS and EB values, as it provides evidence of a favorable dispersion as well as attractive interaction between pure medium-molecular-weight chitosan and CNTs, attributed to the wrapped chitosan. From SEM images, we demonstrated that the coverage of chitosan along carbon nanotubes is clearly visible, exhibiting tube-like structures with diameters between 140 and 300 nm. This is, in turn, associated with the enhanced electrical properties reported for the biopolymer films mainly due to the contacts between the tubes embedded along the chitosan matrix, which contributes to an effective transfer of electrical charge. It was demonstrated through PL analysis that the spectral characteristics of pure chitosan and chitosan/carbon nanotubes were primarily composed of two emission bands. This suggests that the spectral structures are quite similar, indicating that the luminescence is most probably attributed to the chitosan wrapped around CNTs. This research highlights the influence of chitosan molecular weight on the effective wrapping of carbon nanotubes to produce Cs/CNTs film samples with a valuable application, including the field of bioengineering.

## Figures and Tables

**Table 1:** Thickness values of pure Cs and Cs/CNTs films.

Films	Thickness (µm)
LMW-Cs	66
LMW-Cs/CNTs	84
MMW-Cs	54
MMW-Cs/CNTs	66
HMW-Cs	62
HMW-Cs/CNTs	54

**Table 2:** Degree of swelling, solubility in water, and IEC of Cs and Cs/CNTs film samples.

Films	%DS	%SW	IEC (meq g^−1^)
LMW-Cs *	334.5	18.2	0.04
LMW-Cs/CNTs	450.8	23.0	0.057
MMW-Cs *	798.9	18.3	0.041
MMW-Cs/CNTs	517.3	28.4	0.047
HMW-Cs *	747.0	18.3	0.041
HMW-Cs/CNTs	665.0	28.4	0.047

* Data extracted from reference [6].

**Table 3:** Crystallinity index of pure Cs.

Chitosan Molecular Weight	CrI (nm)
LMW-Cs	0.58
MMW-Cs	0.60
HMW-Cs	0.62

**Table 4:** Calculated values for crystallite size, dislocation density, and microstrain of composites film samples.

	Composite Films		
	LCS	MCS	HCS
Crystallite Size (nm)	17.73	39.07	15.12
Microstrain (1 × 10^−3^)	9.27	18.16	7.05
Dislocation density (1 × 10^−2^)	0.43	1.36	0.47

**Table 5:** XPS core-level C 1s and N 1 s BE (eV) for pure Cs films and MMW-Cs-CNTs films.

Film	BE (eV)	
	C 1s	N 1s
Pure chitosan film	284.41	398.77
	285.47	399.96
	288.39	
Chitosan–CNTs film	284.44	399.32
	284.95	400.38
	285.8	
	286.23	
	288.23	

**Table 6:** Mechanical performance of pure Cs films and Cs/CNT film samples.

Films	Tensile Strength (MPa)	Elongation-at-Break (%)	Young Modulus (MPa)	Toughness (MJ m^−3^)
LMW-Cs	72.13 ± 20.03	4.75 ± 1.47	3189.59 ± 524.34	2.06 ± 0.99
LMW-Cs/CNTs	45.62 ± 4.67	3.82 ± 1.19	2122.94 ± 664.58	0.86 ± 0.42
MMW-Cs	35.75 ± 2.88	4.75 ± 0.86	1620.60 ± 232.64	1.17 ± 0.32
MMW-Cs/CNTs	51.26 ± 7.31	7.26 ± 3.03	1895.97 ± 564.78	2.55 ± 1.27
HMW-Cs	49.60 ± 1.55	4.02 ± 0.81	2344.19 ± 466.55	1.21 ± 0.65
HMW-Cs/CNTs	56.46 ± 13.51	4.71 ± 1.32	2627.82 ± 805.32	1.78 ± 0.81

**Table 7:** Electrical properties of chitosan films and Cs/CNTs films.

Films	R_s_ (mΩ/Υ)	Resistivity (Ω cm^−1^)	Electrical Conductivity (S cm^−1^)
LMW-Cs	1 × 10^8^	1.22 × 10^8^	5.1 × 10^−8^
LMW-Cs/CNTs	202.9	538.15	0.0048
MMW-Cs	3.9 × 10^7^	6.71 × 10^7^	5.95 × 10^−8^
MMW-Cs/CNTs	116.7	226.91	0.0156
HMW-Cs	6.7 × 10^7^	9.43 × 10^7^	5.16 × 10^−8^
HMW-Cs/CNTs	174.8	301.30	0.0132

## Data Availability

The original contributions presented in the study are included in the article/Supplementary Material. Further inquiries can be directed to the corresponding author/s.

## Supplementary Materials

The following supporting information can be downloaded at https://www.mdpi.com/article/10.3390/polym17070889/s1: Figure S1: UV-vis absorption spectra of Cs and Cs/CNTs liquid solutions of (a) pure chitosan, (b) chitosan-CNTs.; Figure S2: second-derivative of the FTIR spectra of pure chitosan film samples and chitosan-CNTs film samples.; Figure S3: Enlarged comparison of the (004) plane of Cs/CNTs film samples.; Figure S4: XRD pattern of (a, c, e) Cs sample films and (b, d, f) Cs/CNTs sample films.; Figure S5: XPS survey spectrum of MMW-Cs film, and MMW-Cs/CNTs film.; Figure S6: Demonstrating the electrically conductive Cs/CNTs film sample in comparison to the non-electrically conductive chitosan film.; Figure S7: CIE 1931 coordinates of the film samples of pure chitosan and composites films.; Table S1: Color attributes and opacity values of the film samples. Table S2: Color purity values of the Cs and Cs-CNTs composite films. References [17,60,61,62,63,64,65] are cited in the supplementary materials.

## Abbreviations

The following abbreviations are used in this manuscript:

Cs	Chitosan
CNTs	Carbon nanotubes
LMW-Cs	Low-molecular-weight chitosan
MMW-Cs	Medium-molecular-weight chitosan
HMW-Cs	High-molecular-weight chitosan
LMW-Cs/CNTs	Low-molecular-weight chitosan/carbon nanotubes
MMW-Cs/CNTs	Medium-molecular-weight chitosan/carbon nanotubes
HMW-Cs/CNTs	High-molecular-weight chitosan/carbon nanotubes
