# Supplementary material for: Study of the Influence of Chitosan-Wrapped Carbon Nanotubes on Biopolymer Film Properties
Source: Polymers (Basel). 2025 Mar 26;17(7):889. doi: 10.3390/polym17070889 (PMC11991639; doi:10.3390/polym17070889)
Supplement: Supplementary file 1 [file polymers-17-00889-s001.zip › polymers-3518081-supplementary.pdf]

Study of the Influence of Chitosan-Wrapped Carbon Nanotubes on Biopolymer Film Properties

Aurora G. Magallanes-Vallejo [1], Ana B. López-Oyama [2,3,*], Eugenio Rodríguez González [1,*], Deyanira Del Angel-López [1], Eder U. Pulido-Barragán [1], Crescencio García-Guendulain [4], Tomás J. Madera-Santana [5], César Rodríguez-Beas [6] and Rogelio Gámez-Corrales [6]

[1] Instituto Politécnico Nacional, Centro de Investigación en Ciencia Aplicada y Tecnología Avanzada-Unidad Altamira, Km 14.5 Carr, Tampico-Puerto Industrial, Altamira 89600, Tamaulipas, México; aumagallanes@gmail.com (A.G.M.-V.); ddelangel@ipn.mx (D.D.Á.-L.); eder.pulido.bar@gmail.com (E.U.P.-B.)

[2] Departamento de Investigación en Física (DIFUS), Universidad de Sonora, Blvd. Transversal S/N, Hermosillo 83000, Sonora, México

[3] Secihti-DIFUS, Universidad de Sonora, Blvd. Transversal S/N, Hermosillo 83000, Sonora, México

[4] School of Engineering and Science, Tecnologico de Monterrey, Mexico City 14380, Mexico;
crescencio.garcia@tec.mx

[5] Centro de Investigación en Alimentación y Desarrollo, A.C., Carr. Gustavo E. Astiazarán Rosas No. 46, Col. La Victoria, Hermosillo 83304, Sonora, Mexico; madera@ciad.mx

[6] Departamento de Física, Universidad de Sonora, Blvd. Transversal S/N, Hermosillo 83000, Sonora, México; cesar.rodriguez@unison.mx (C.R.-B.); rogelio.gamez@unison.mx (R.G.-C.)

* Correspondence: ablopezoy@secihti.mx (A.B.L.-O.); eugenior62@gmail.com (E.R.-G.)

I. Supporting Methods

Characterization of Cs and Cs-CNTs liquid solutions

1. UV-Vis spectroscopy

Uv-Vis spectra of aqueous solutions were acquired using a Perkin Elmer Lambda 45 dual beam spectrometer at a scan rate of 280 nm/min within the range of 200-900 nm. The samples were dissolved in acetic acid and allowed to settle for one hour at a final volume of 3 mL.

Characterization of Cs and Cs-CNTs film samples

1. *Color analysis*

The film color analyses were conducted using a Minolta Chroma Meter colorimeter CR 300 (Osaka, Japan). The use of the CIELAB scale was employing in determining color parameters, specifically the lightness scale from black to white (L), and the chromaticity parameters, $a*$ (redness-greenness) and $b*$ (yellowness-blueness). All measures were carried out on a standard white plate. Six replicates for each sample were measured. The calculation of color differences (ΔE) was obtained as follows:

$$\Delta E = [(\Delta L^*)^2 + (\Delta a^*)^2 + (\Delta b^*)^2]^{1/2} \quad \text{(S2)}$$

$$\Delta L^* = L^* - L_0,\ \Delta a = a^* - a_0 \text{ and } \Delta b^* = b^* - b_0 \quad \text{(S3)}$$

L^*, a^*, and b^*, represent the standard color parameters, while L_0, a_0, and b_0 represent the color parameter of the sample.

2. *Films Transparency*

Films transparency was calculated in accordance to the procedure described by Vega et al. [60]. Each sample was cut into a square shape 81.25 x 1.25 cm) and placed on the interior section of a spectrophotometer cell (Agilent Technologies Cary 5000 UV-vis NIR) that scanned within the 200-900 nm in range. The measured absorbance at 800 nm (A_{800}) was employed for transparency calculation according to Han et al. [61] equation:

$$Transparency = \frac{A_{800}}{T} \quad \text{(S1)}$$

A_{800} is the measure of absorbance at a wavelength of 800 nm, whereas T stands for the thickness of the film in millimeters. To obtain an average value, each film was measured three times.

Results

UV-Vis analysis of Cs and Cs-CNTs liquid solutions

Chitosan and CNT/chitosan solutions were subjected to UV-vis spectroscopy analysis in acetic acid (0.001 mg mL^{-1}) in the wavelength range of 200-900 nm to enable the characterization of absorption features of homogeneous dispersions. UV-vis absorbance spectra of pure chitosan and chitosan-CNT solutions are shown. Fig. 1 (a) depicts the absorbance spectrum of an aqueous solution of pure LMW-Cs, MMW-Cs, and HMW-Cs. The 228 and 283 nm wavelengths within the 400-200 nm region display significant absorption, as shown by the spectra. The characteristic peak of pure chitosan at ca. 228 nm and the increase in absorption near to 240 nm are linked to the decrease in molecular weight, which is in accordance with the conclusions of other researchers. The observed wavelength displacement was attributed to a change in the molecular weight of the polymer. The ionic linkages ($\pi \rightarrow \pi$) are believed to be responsible for the peak observed at 228 nm in the UV-vis spectrum. As per other authors, the absorption near 280 nm is linked to unsaturated bonds of covalent linkages ($\eta \rightarrow \pi$), primarily C-O, which leads to an increase in the absorption intensity with a decrease in molecular weight [62,63]. LMW-Cs-CNTs, MMW-Cs-CNTs, and HMW-Cs-CNT liquid solutions displayed the typical absorption peaks at 229 and 288 nm, respectively (Fig. 1 (b)), which could be associated with the ability of chitosan to wrap along the CNT surface. Due to its hydrophobic behavior, the pristine CNTs are weak to form stable liquid solutions in water, which is necessary for steady characterization using UV-vis spectroscopy. In accordance with Rodríguez et al., [17] the use of acetic acid is associated with improved stability of carbon nanotube liquid solutions and an enhanced signal-to-noise ratio. The acid-dispersed CNTs formed stable solutions up to 0.001 mg mL^{-1} concentration without solubilizing agents, confirming that all the spectroscopic signals detected during our experiments correspond to the CNT. The range of 228-232 nm has been found to exhibit maximum absorption in the UV-visible range, which is consistent with previous research [64].(Rance et al. 2010) According to John et al., [65] the broadness of the peak indicates that the absorbance occurred across a wide distribution of absorbing units, which could be associated with distinct levels of order within the polymeric network.

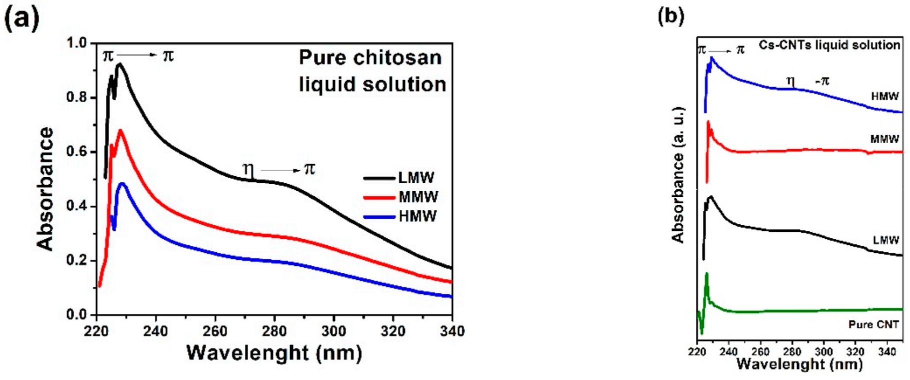

Figure S1. *UV-vis absorption spectra of Cs and Cs-CNTs liquid solutions of (a) pure chitosan, (b) chitosan-CNTs.*

Color attributes analysis of Cs and Cs-CNTs film samples
Color and opacity of the films

The film samples color values are shown in Table S1. The brightness of the film is indicated by the *L** value. On the other hand, it is important to note that *a** denotes greenness/redness, while *b** represents blueness/yellowness in the test films. Pure chitosan films displayed yellow color. There is no significant difference in the *L** parameters of chitosan from LMW-Cs to HMW-Cs. Based on the analysis of the *L** parameter, it is evident that CNT played a role in the increase in opacity, leading to a decrease in luminosity. This increase in opacity is attributed to the rise in black-natural carbon-based materials, leading to a reduction in the intrinsic yellow color of the chitosan in chitosan-CNT film samples. According to Table S1, the total color difference between chitosan and chitosan-CNT film samples was observed to fluctuate between 8.70 and 67.85.

The *L*-value of chitosan films remained constant, regardless of their molecular weight. However, a significant reduction in the *L*-value was observed in the chitosan-CNT films. From *L*-parameter values, the Chitosan-CNTs film samples showed more browning color. LMW-Cs-CNTs film chitosan was the darkest one. The efficiency of the light absorption process is improved through the application of the carbon nanotube structure. The creation of voids through the CNT's rod-like structure enables the absorption of incident light, ultimately aiding the deep targeting of light within the interstitial regions of the CNT. This facilitates the achievement of light absorption in chitosan-CNT films.

Film transparency depends on the crystallinity of the polymeric lattice. The correlation between XRD and color results revealed that an increase in crystallinity leads to a decrease in transparency (or an increase in opacity) of the polymer due to a corresponding increase in density. The addition of carbon-based materials was found to increase the crystallite size of chitosan-CNT films. Non-functionalized CNTs has a high aspect ratio and strong aggregation, due to the van der Waals attraction, which leads to complex networks that contribute to the crystallite size increase [64].

Table S1. Color attributes and opacity values of the film samples

Films	***Color***			**ΔE**	**Opacity ($A \cdot mm^{-1}$)**
	L*	***a****	***b****		
**LMW-Cs**	86.8±0.35	-1.13±0.11	6.83±0.56	12.08 ± 0.56	5.74
**LMW-Cs-CNT-**	30.52 ± 0.24	-0.23 ± 0.03	-0.20 ± 0.06	67.17 ± 0.23	0.8
**MMW.-Cs**	87.66±0.68	-0.27±0.07	1.59±0.31	10.01± 0.67	7.01
**MMW-Cs-CNT**	29.84 ± 2.12	-0.26 ± 0.05	-0.13 ± 0.17	67.85 ± 2.11	0.25
**HMW-Cs**	89.45±0.94	0.20±0.08	-1.20±0.30	8.70 ± 0.89	6.11
**HMW-Cs-CNT**	30.18 ± 2.04	-0.21 ± 0.01	-0.09 ± 0.17	67.51 ± 2.03	1.59

Table S2. Color purity values of the Cs and Cs-CNTs composite films

Films	*Color purity (%)*
LMW-Cs	16
MMW-Cs	*14*
HMW-Cs	*13*
LMW-Cs-CNTs	*5*
MMW-Cs-CNTs	*2*

II. Supporting Figures and Tables

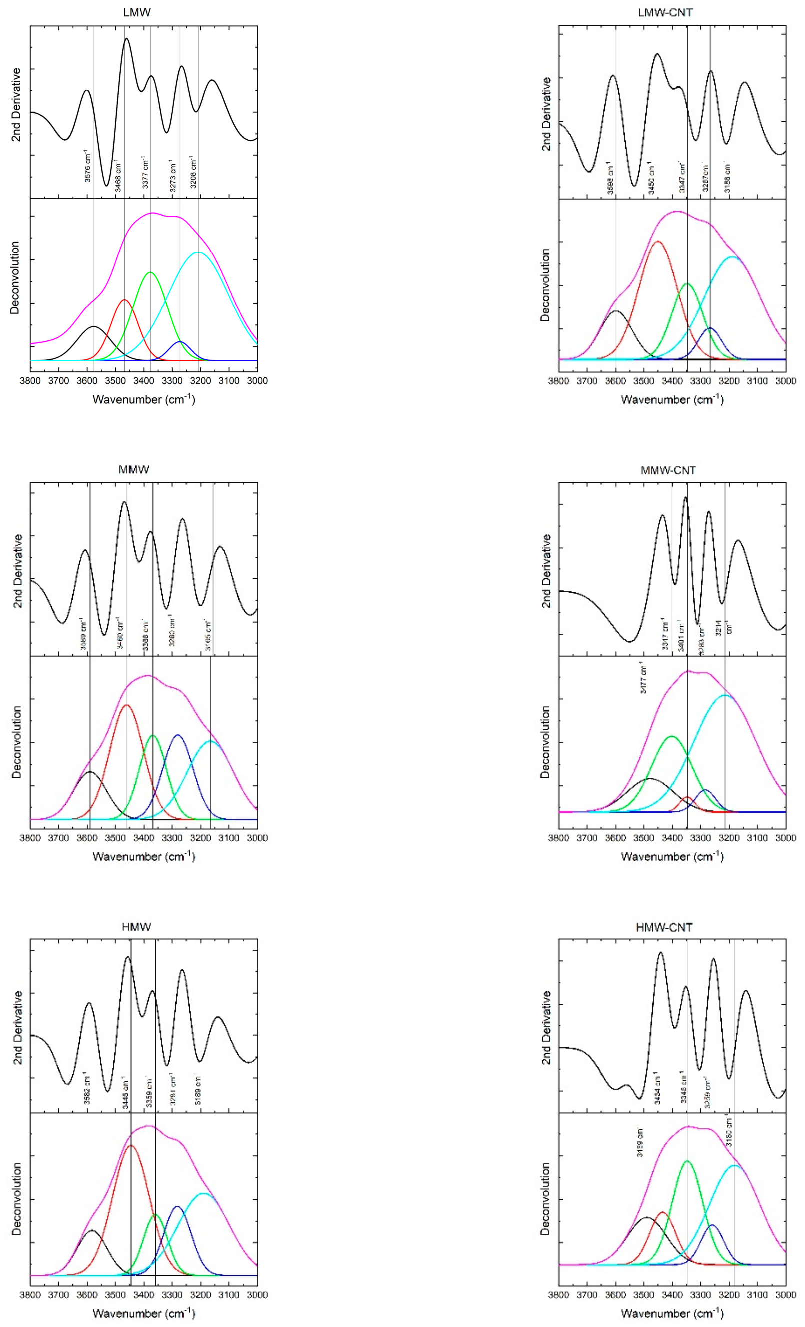

Figure S2. Second-derivative of the FTIR spectra of pure chitosan film samples and chitosan-CNTs film samples

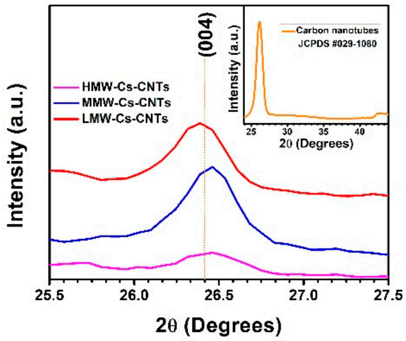

Figure S3. Enlarged comparison of the (004) plane of (a) Cs-CNTs film samples

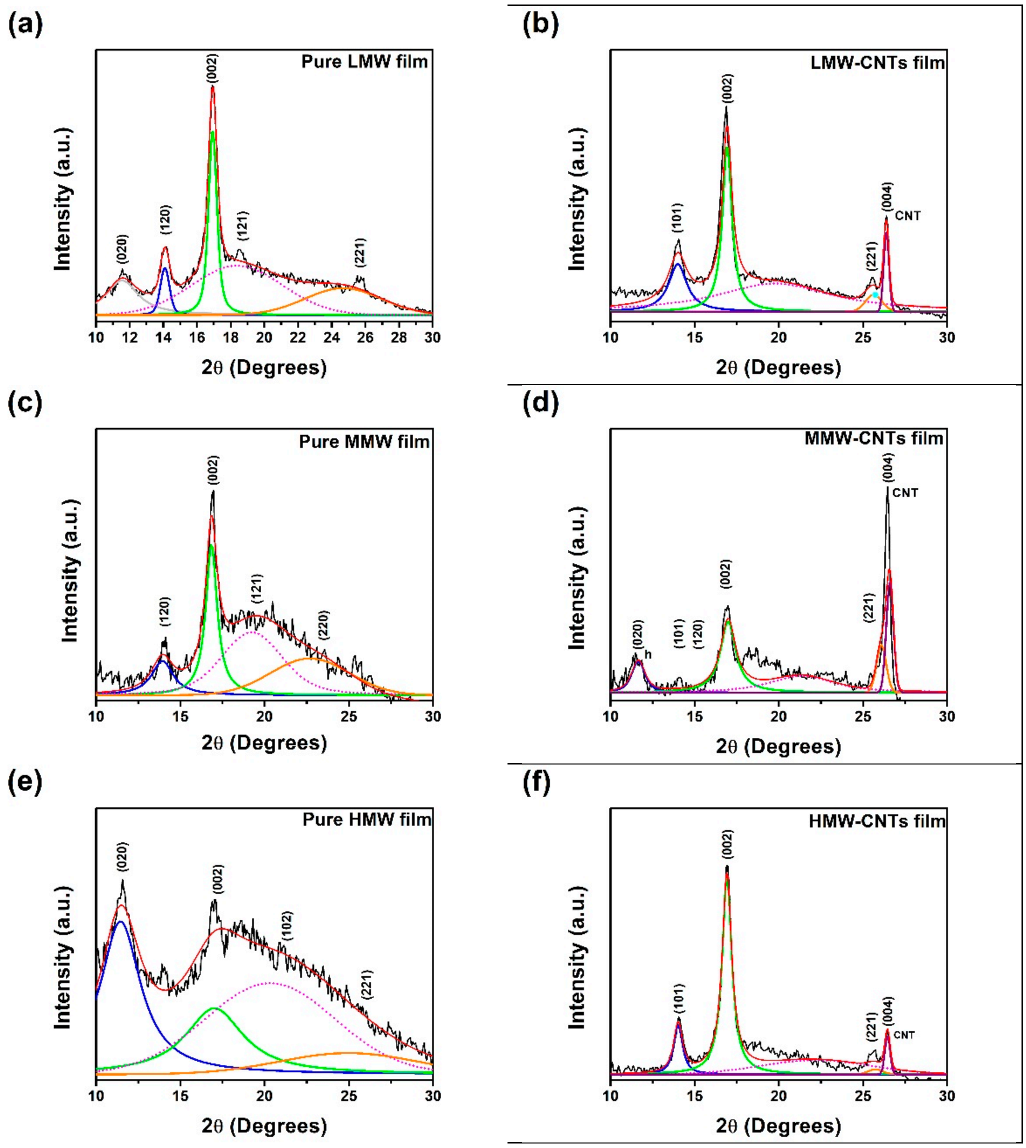

Figure S4. XRD pattern of (a, c, e) Cs sample films and (b, d, f) Cs-CNTs sample films

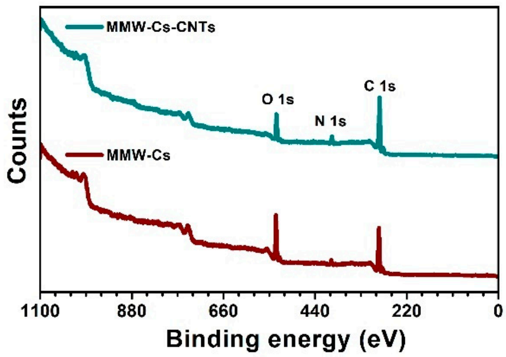

Figure S5. XPS survey spectrum of MMW-Cs film, and MMW-Cs-CNTs film.

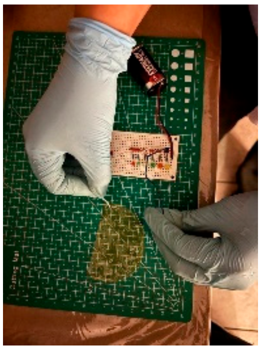

Pure chitosan film

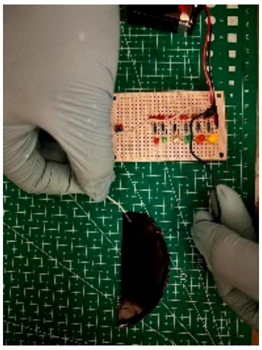

Chitosan-CNTs film

Figure S6. Demonstrating the electrically conductive Cs-CNTs film sample in comparison to the non-electrically conductive chitosan film.

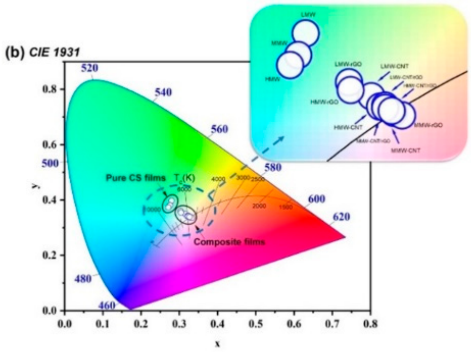

Figure S7. CIE 1931 coordinates of the film samples of pure chitosan and composites films
